# Data on morphological features change of pre-hydrolysis treated sugarcane bagasse using in-situ sodium hydroxide-sodium bisulfate method

**DOI:** 10.1016/j.dib.2019.103971

**Published:** 2019-05-07

**Authors:** Abdel-Naser Zohri, Mohamed Abdelwahab, Maysa Ali, Sara Ibrahim, Mohamed Abdelazim

**Affiliations:** a Botany and Microbiology Department, Faculty of Science, Assiut University, 71516, Egypt; b Chemistry Department, Faculty of Science, Assiut University, 71516, Egypt; c Chemical and Biotechnological Laboratories, Assiut University, Sugar Industry Technology Research Institute, 71516, Egypt

**Keywords:** Biomass, Pretreatment, In-situ, Cellulose, Enzymatic hydrolysis, Biofuel

## Abstract

The Scan Electron Microscope Images (SEM), X-ray Diffraction and Fourier Transform Infrared Spectroscopy (FTIR) dataset has been outlined investigating morphological features change of native sugarcane bagasse, as an agro-industrial lignocellulosic feedstock waste and a potential for cellulose biopolymer extraction, pretreated by alkali (sodium hydroxide) followed by an acid step (sodium bisulfate) in an exothermic in-situ one step, pretreated by acid (sulfuric acid) followed by residual solid fraction alkali pretreatment (sodium hydroxide) in a two separate individual steps and finally after the enzymatic cellulolysis. Data explained herein helps to extend and add to knowledge regarding the impact unlikeness of two different pretreatment methodologies utilize the same chemicals and relatively same concentrations on the cellulosic fiber morphological features and consequently its enzymatic accessibility. This data are related to Egypt Patent Office application, 1349/2017, entitled “In-situ sodium hydroxide-sodium bisulfate sugarcane bagasse pretreatment for biofuel production”,Zohri et al., 2017 [1].

**Table:** Specifications table

Subject area	*Chemistry, biomass, Microbiology and Biofuel.*
More specific subject area	*Physiochemical Biomass Pretreatment for Biofuel.*
Type of data	*X-ray, SEM, FTIR, Tables and Text files.*
How data was acquired	*Microscope, SEM (JOEL-JSM* 5400 LV*), X-ray diffractometer(Philips PW 1710), FTIR(Nicollet 6700)*
Data format	*Raw, filtered and analyzed.*
Experimental factors	*SEM, fine powder was dried and coated with gold and was imaged.* *X-ray, dry fine powder.* *FTIR, dry fine powder-containing potassium bromide tablet.*
Experimental features	*SEM, an energetic electron beam bombards biopolymer surface.* *X-ray, X-ray bombards biopolymer surface.* *FTIR, Infra-Red light passes through biopolymer surface.*
Data source location	*Assiut city, Upper Egypt, 357 Kilometers south of capital Cairo, 71516, EGYPT.*
Data accessibility	*Egypt Patent Office, 1349/2017, Abdel-Naser Zohri, Mohamed Abdelwahab, Maysa Ali, Sara Ibrahim, Mohamed Abdelazim, In-situ Sodium hydroxide-Sodium Bisulfate Sugarcane Bgasse Pretreatment for Biofuel Production.*
Related research	*Abdel-Naser Zohri, Mohamed Abdelwahab, Maysa Ali, Sara Ibrahim, Mohamed Abdelazim, In-situ Sodium hydroxide-Sodium Bisulfate Sugarcane Bgasse Pretreatment for Biofuel Production, Egypt Patent Office, 1349/2017.*

**Table:** 

**Value of the data** • A convenient alkali-acid in-situ biomass pretreatment. • A potential for cellulose-lignin valorization. • Applicable to a variety of lignocellulosic feedstocks, rice straw and sweet sorghum. • Easily commercialized in case of chemical recovery investigation. • Essential for biorefinery concept. • High glucose yield employing low cellulytic enzyme load.

## 1 Data

### 1.1 Scan electron microscopy, SEM micrographs

#### 1.1.1 Native bagasse

**Fig. 1:**
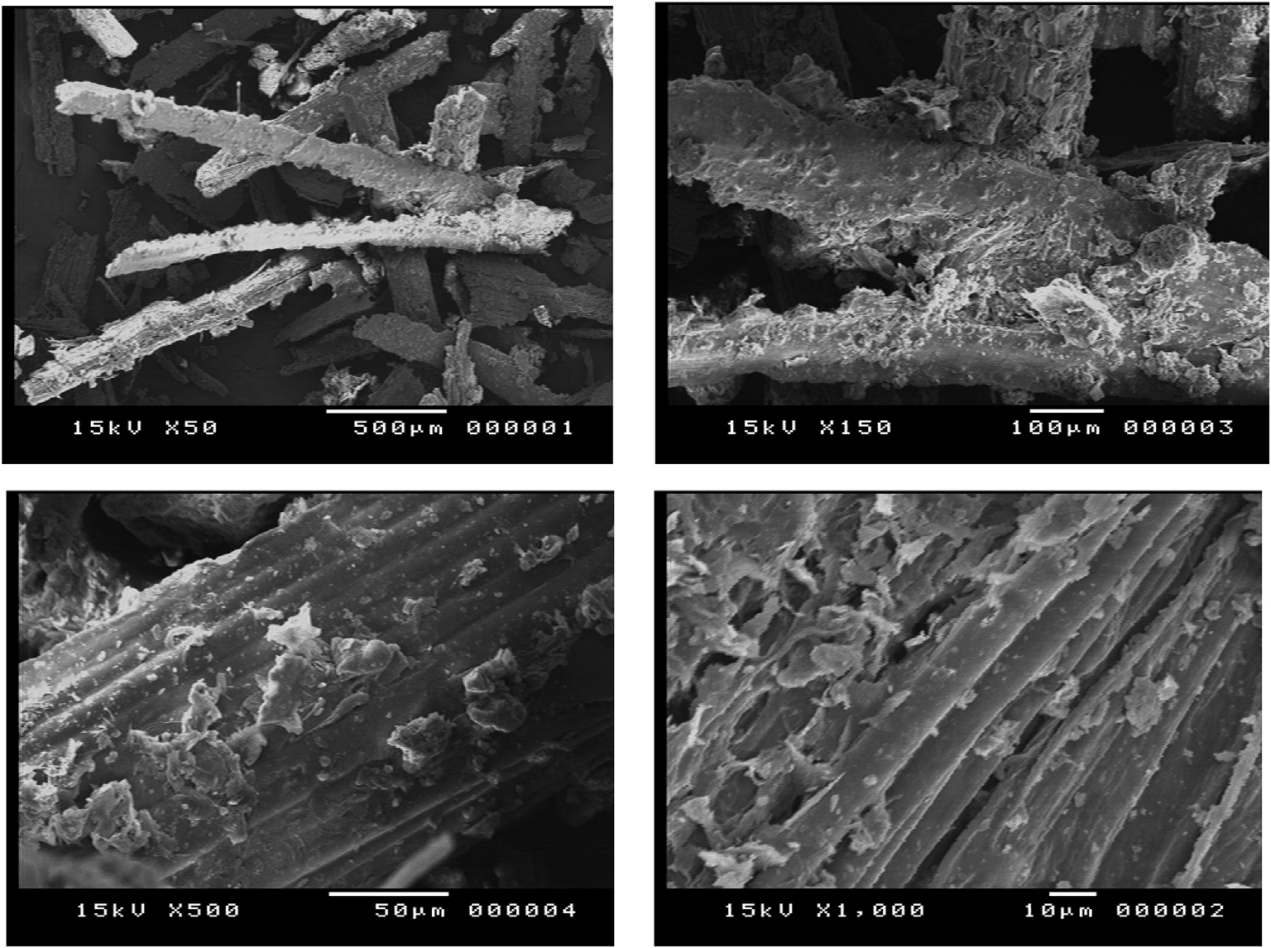
SEM Micrographs of native sugarcane bagasse.

Fig. 1 shows the compact structure of native sugarcane bagasse. Briefly the compact structure due to the interconnection between cellulose and hemicelluloses biopolymers imbedded in lignin structure showed in four different magnifications.

#### 1.1.2 Sugarcane bagasse pretreated by in-situ process (alkali/acid one step)

**Fig. 2:**
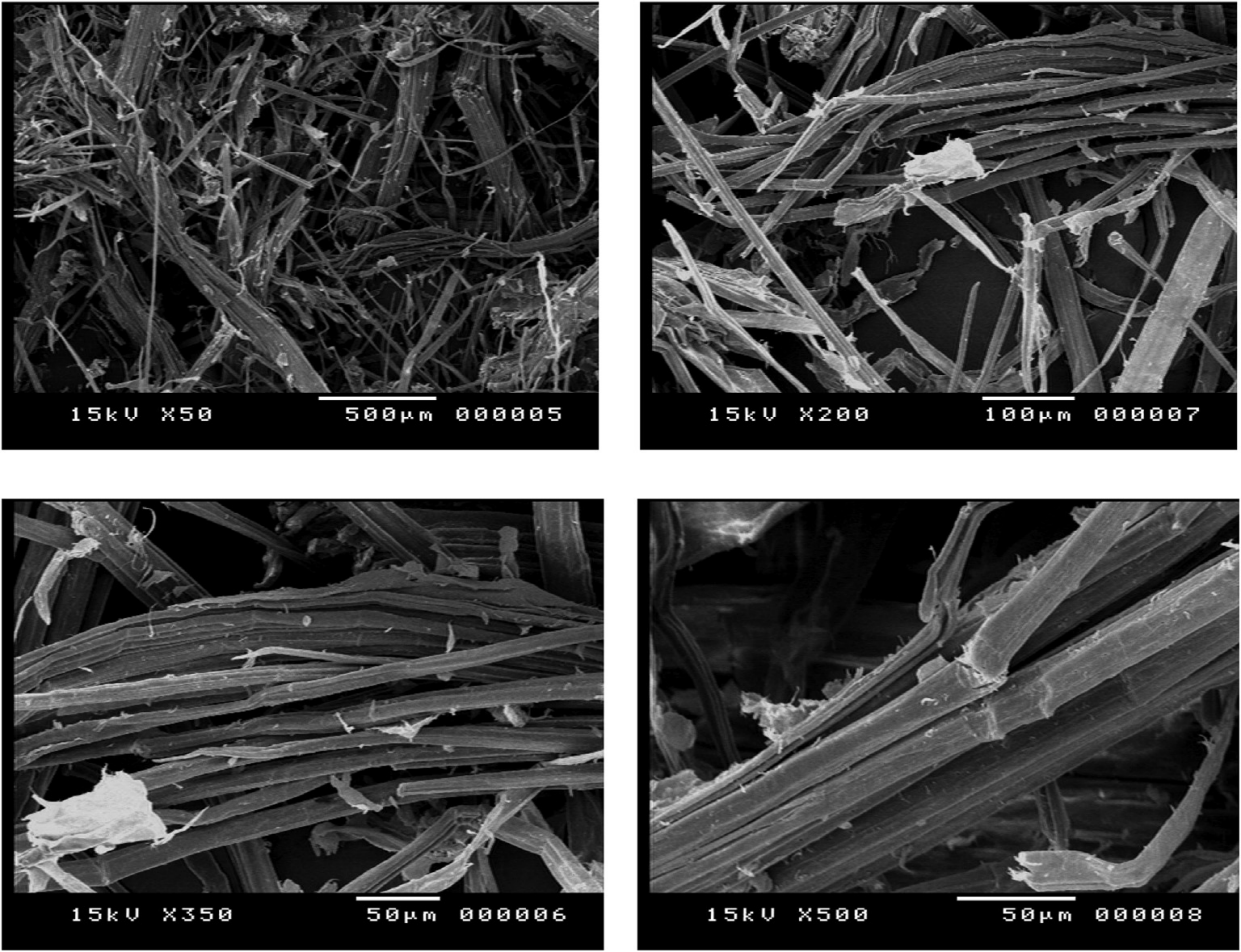
SEM Micrographs of In-situ process pretreated sugarcane bagasse.

Fig. 2 represents the solid residual fraction after the In-situ pre-hydrolysis treatment step shows the deconstruction of the compact rigid structure into cellulose fibers appeared in irregular uniform long strips reveals disaggregated cellulose release from lignocellulosic matrix as a loosen bundles.

#### 1.1.3 Sugarcane bagasse pretreated by acid followed by alkali (a two separate steps)

**Fig. 3:**
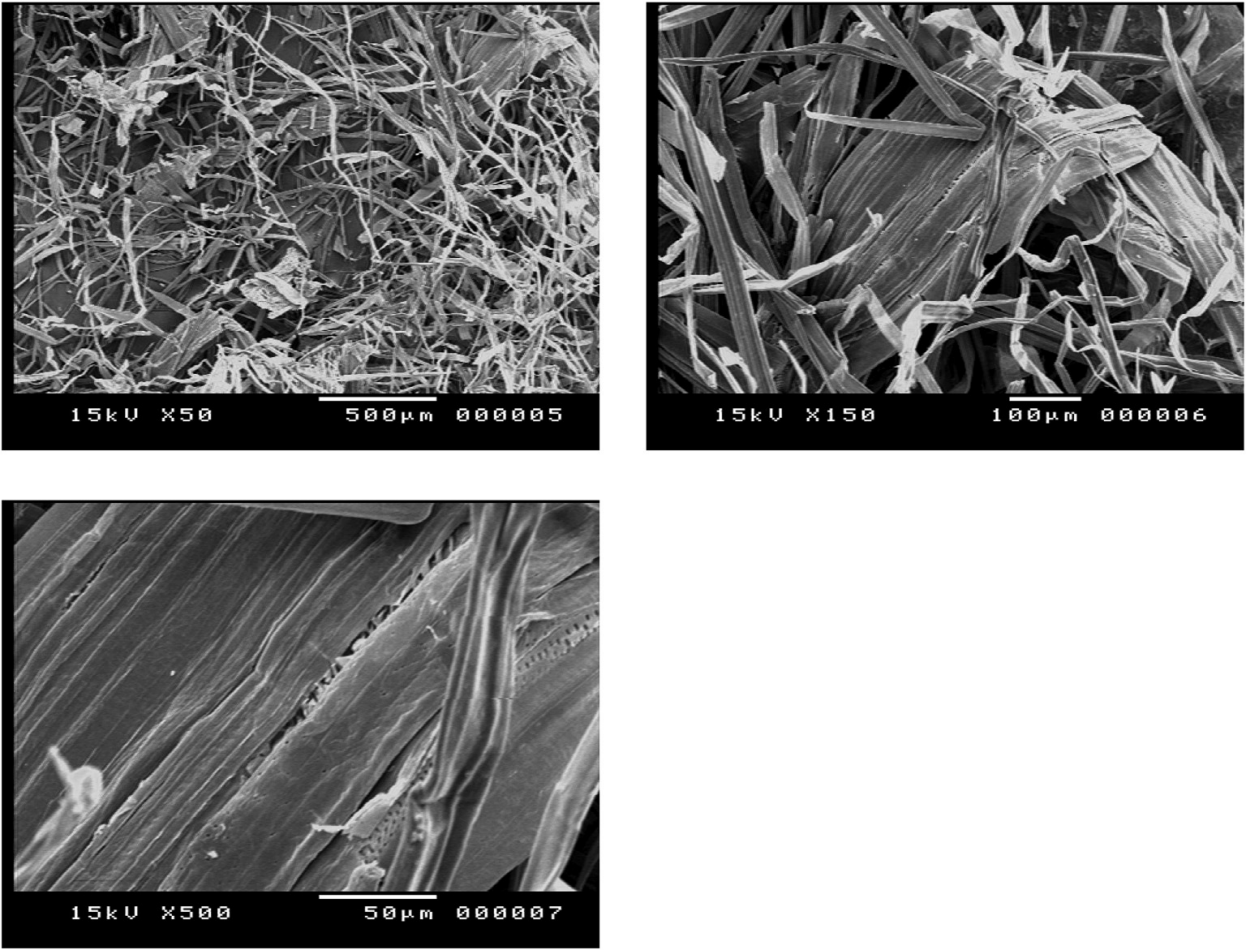
SEM Micrographs of sugarcane bagasse pretreated by acid followed by alkali in a two separate steps.

Fig. 3 shows the impact of acid pretreatment of sugarcane bagasse and the resulting solid fraction was subjected to alkali pretreatment in a two individual separate steps, it is relatively similar to Fig. (2) in exception of still cellulose fibers remains aggregated and tight, released from the rigid compact structure and some cellulose bundles still attached to each other.

#### 1.1.4 In-situ process pretreated sugarcane bagasse morphology after the enzymatic cellulolysis

**Fig. 4:**
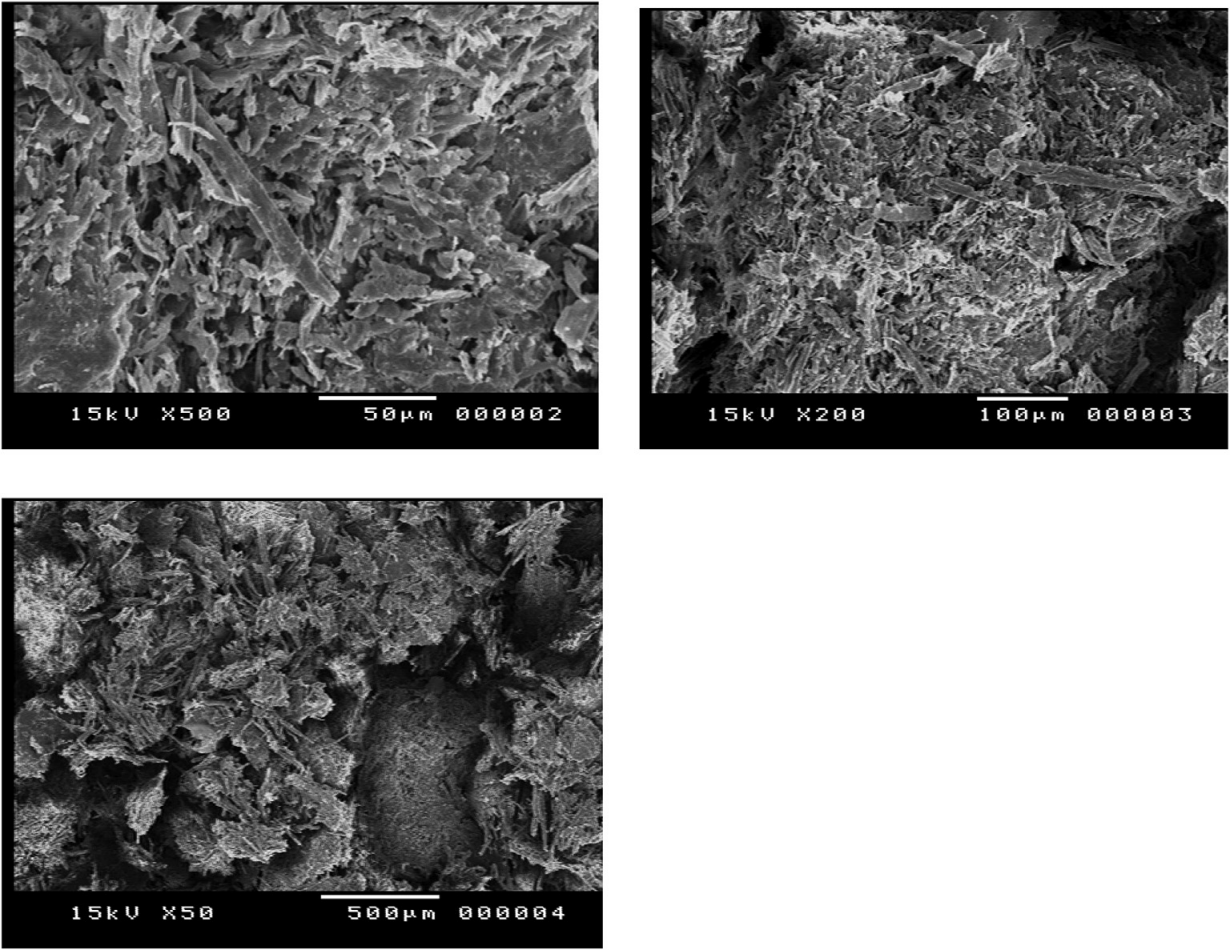
SEM Micrographs of solid residual remained after enzymatic hydrolysis.

Fig. 4 shows the disappearance of the elongated disaggregated cellulose fibers due to the action of cellulase enzymes leads to soluble reducing sugar release, the micrographs of the remaining solid fraction after hydrolysis shows the amorphous morphology of the residual lignin and hemicellulose biopolymers-containing substrate after pretreatment and still cover a low percent of cellulose fibers that were not hydrolyzed under the action of the cellulytic enzymes.

### 1.2 X-ray diffraction analysis, XRD diffractograms

#### 1.2.1 1*. X-ray diffractogram of native bagasse*

**Fig. 5:**
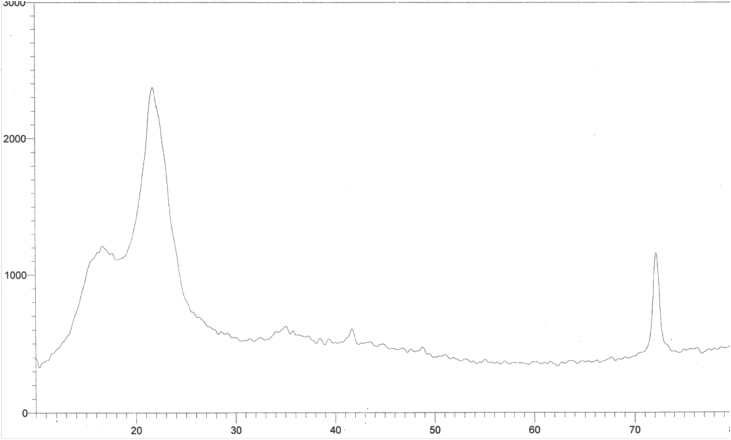
X-ray Diffractogram of native bagsse.

Fig. 5 shows the higher peak intensity at 2theta 23 as well as the higher peak minima at 2theta 18.5 gives overall lower crystallinity index 53.3% due to the presence of high percentage of amorphous lignin and hemicelluloses in combination with the crystalline cellulose polymer.

#### 1.2.2 X-ray diffractogram of in-situ pretreated sugarcane bagasse

**Fig. 6:**
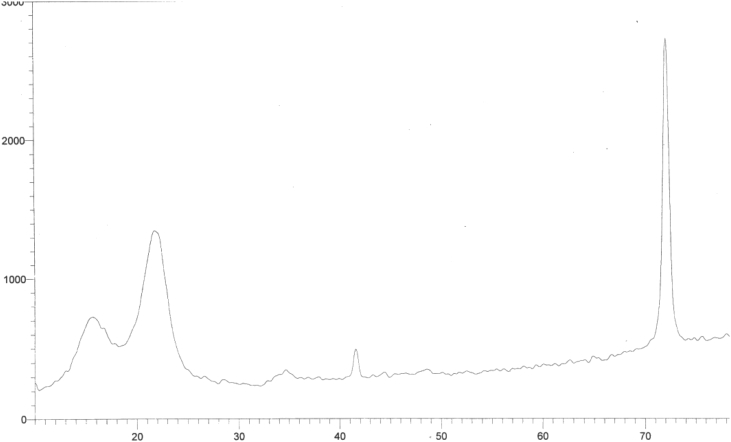
X-ray Diffractogram of In-situ pretreated sugarcane bagasse.

Fig. 6 shows a lower peak intensity at 2theta 23 as well as a lower peak minima at 2theta 18.5 gives overall lower crystallinity index 62.5% as compared with its high cellulose portion 83.4% and lower lignin and hemicelluloses content indicates relatively the amorphous structure of the resulting In-situ substrate.

#### 1.2.3 X-ray diffractogram of acid/alkali pretreated sugarcane bagasse in a two separate steps

**Fig. 7:**
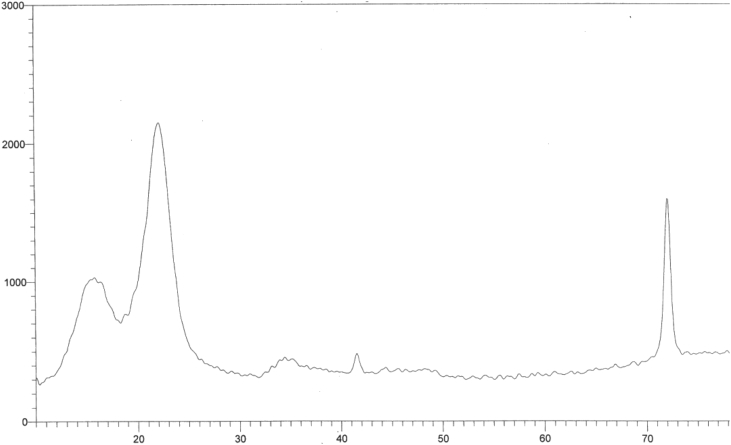
X-ray Diffractogram of acid/alkali pretreated sugarcane bagsse in a two separate steps.

Fig. 7 shows the higher peak intensity at 2theta 23 and higher peak minima at 2theta 18.5 gives overall high crystallinity index 68.7% as compared with the In-situ substrate and relatively their same cellulose content.

### 1.3 Fourier Transform Infrared Spectroscopy, FTIR spectrograms

#### 1.3.1 Native bagasse FTIR spectrogram

**Fig. 8:**
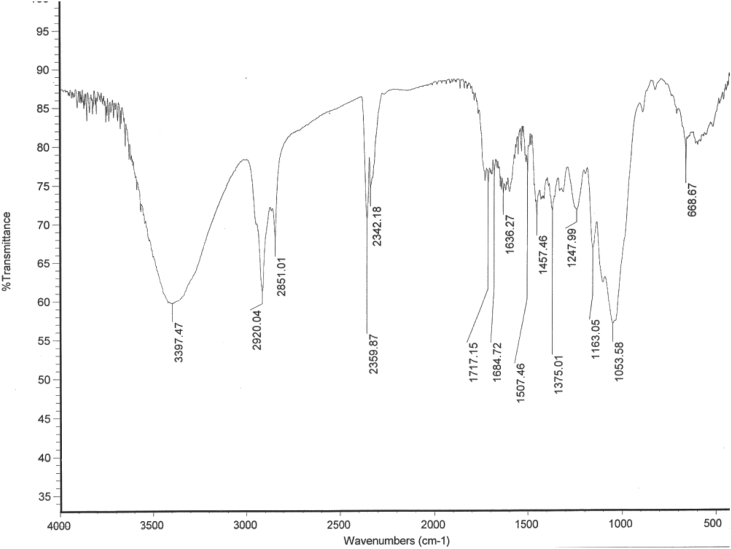
FTIR Spectrogram of native bagasse.

Fig. 8 shows the Infra-red spectrum of native bagasse indicates the presence band at 1717 cm^−1^ belongs to acetyl content of hemicelluloses and low peak intensity at 3397 cm^−1^ belongs to cellulose aliphatic hydroxyl content due to the buried cellulose fibers in the compact lignocellulosic structure.

#### 1.3.2 FTIR spectrogram of in-situ pretreated sugarcane bagasse

**Fig. 9:**
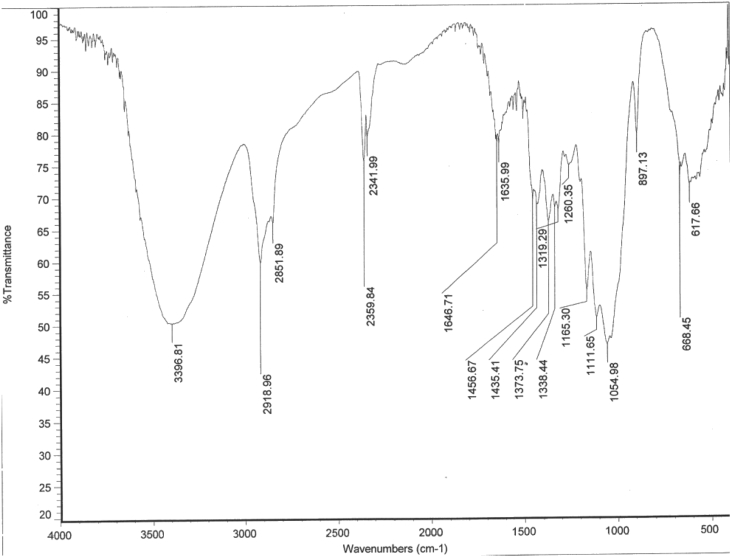
FTIR Spectrogram of In-situ pretreated sugarcane bagasse.

Fig. 9 shows the disappearance of peak at 1717 cm^−1^ and the high intensity of the peak at 3397 cm^−1^ due to release of cellulose bundles from the compact aggregated lignocellulosic matrix.

## 2 Experimental design, materials and methods

### 2.1 Sugarcane bagasse

Sugarcane bagsse was provided by Abo-Korkas sugarcane milling factory, Elminia Governorate, Egypt, during the season 2015–2016. Chipped into small pieces 1 cm length, washed several times with water and stored in refrigerator prior to the pretreatment process.

### 2.2 Pretreatment

Pretreatment was carried out in autoclave, Systec VE-75. using the procedures described In-situ sodium hydroxide-sodium bisulfate [1] as well as acid pretreatment [2] followed by the resulting solid fraction alkali pretreatment [3].

#### 2.2.1 Alkali/acid in-situ process

8 gm sugarcane bagasse, 1–2 cm length, immersed in 80 ml sodium hydroxide 1.3% and autoclaved at 120 °C for 40 min. Then a 20 ml water containing 1.45 ml sulfuric acid 98% is added while the slurry temperature 80 °C and the temperature kept at 105 °C for further 40 min. The resulting solid fraction was washed with water and post treated by 0.2% sodium hydroxide dissolution in cold for lignin recovery and cellulose fiber separation, solid yield 47.5%.

#### 2.2.2 Acid/alkali two separate individual steps

10 gm sugarcane bagasse, 1–2 cm in length, immersed in 100 ml sulfuric acid 1% and autoclaved at 120 °C for 40 min. The resulting solid fraction was filtered and washed with a plenty of water and dried.

Dry residual solid fraction was immersed in sodium hydroxide solution 2%, 1:10 solid to liquid ratio and autoclaved at 120 °C for 40 min, solid yield 28.3%.

### 2.3 Scan electron microscopy

Bagasse morphology was analyzed before and after pretreatment in addition to after enzymatic hydrolysis. Samples were dried and coated with gold and were imaged using scan electron microscopy, JOEL-JSM 5400 LV (Japan).

### 2.4 X-ray diffraction

X-ray diffraction data were obtained in Philips PW 1710 using monochromatic CuKα radiation (1.54 Å), 40 KV and 30 mA setting. The crystallinity index for all the samples was calculated according to the procedure proposed in [4], [5].

### 2.5 Fourier transform infrared analysis

FTIR spectra were recorded on Nicollet 6700 spectrophotometer using potassium bromide.

### 2.6 Enzymatic hydrolysis and fermentation

Enzymatic hydrolysis of pretreated sugarcane bagasse was carried out at a substrate ratio of 2.5% (wt/v) in 0.05 M sodium citrate buffer (pH 5.0), at 45 °C. The cellulase applied consist of 12 filter paper units of Cellic Ctech2, 0.12 ml, per 1 g solid substrate (Novozymes, Denmark). The hydrolysis yield expressed in glucose yield was determined as described by Maeda et al. [6] and its relation to total reducing sugar released was determined by procedures by Zohri et al. [7].

Fermentation was carried out using single batch SSF, Separate Hydrolysis and Fermentation, utilizes fed-batch enzymatic hydrolysis at 15% water solid load, centrifuging then collecting the clear sugar solution nutrient enriched with 5 g/l peptone, 3 g/l yeast extract, 3 g/l malt extract and 1 g/l Saccharomyces cerevisiae affording 78.2% fermentation efficiency.

The following Table 1, represents a comparison of some important parameters between In-situ pretreatment and two individual acid/alkali separate steps and it has been deposited in this laboratory [8] records as a novel important coupled acid and base sugarcane bagasse pretreatment necessary to overcome its high hemicellulose acetyl content and lignin content recalcitrant its enzymatic hydrolysis.

**Table 1:** Comparison between in-situ and the separate two individual processes.

	In-situ process	Two separate acid/alkali steps
Methodology	One-pot	Individual two separate steps
Sodium hydroxide conc.	1.3%	2%
Sulfuric acid conc.	1.45%, acid assay 98%	1%, acid assay 98%
Water input	1:12.5 solid to water ratio (overall process)	1:17 solid to water ratio (overall process)
Heat demand	Exothermic, second in-situ step	Endothermic, both two steps
Cellulose loss	5.8%	33.4%
Fiber morphology	Disaggregated, loosen bundles	Aggregated, tight bundles
Cellulase enzyme load, Name	0.12 ml, 12 FPU, Cellic Ctech2 per 1 g substrate.	0.4 ml, 25 FPU, Accelerase 1500 and 50 FPU, Novozyme 188 per 1 g substrate, [3].
Hydrolysis yield%	75.4%	72.3%
Hydrolysis time, h.	48 h.	72 h.
