# Supplementary material for: Data on morphological features change of pre-hydrolysis treated sugarcane bagasse using in-situ sodium hydroxide-sodium bisulfate method
Source: Data Brief. 2019 May 7;24:103971. doi: 10.1016/j.dib.2019.103971 (PMC6525299; doi:10.1016/j.dib.2019.103971)
Supplement: Multimedia component 1 [file mmc1.docx]

Author Agreement

I am hereby grants the Journal of advanced research full and exclusive rights to the manuscript, all revisions, and the full copyright. The Journa rights include but are not limited to the following:

(1) to reproduce, publish, sell, and distribute copies of the manuscript, selections of the manuscript, and translations and other derivative works based upon the manuscript, in print, audio-visual, electronic, or by any and all media now or hereafter known or devised;

(2) to license reprints of the manuscript to third persons for educational photocopying;

(3) to license others to create abstracts of the manuscript and to index the manuscript;

(4) to license secondary publishers to reproduce the manuscript in print, microform, or any computer-readable form, including electronic on-line databases.

(5) to license the manuscript for document delivery. These exclusive rights run the full term of the copyright, and all renewals and extensions thereof. I hereby accept the terms of the above Author Agreement and I sign on behalf of the authors.

**Also I declare that all authors have no conflict of interest.**

Yours sincerely

Mohamed Abdelazim Abulela
