# Supplementary figures and images for: Data on morphological features change of pre-hydrolysis treated sugarcane bagasse using in-situ sodium hydroxide-sodium bisulfate method
Source: Data Brief. 2019 May 7;24:103971. doi: 10.1016/j.dib.2019.103971 (PMC6525299; doi:10.1016/j.dib.2019.103971)

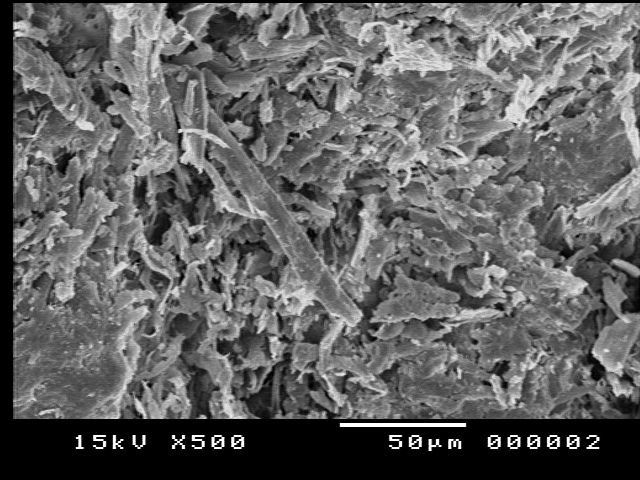

Supplement: Supplementary file 2 — Multimedia component 2 [file mmc2.zip › dib_103971_1_mmc2.bmp]

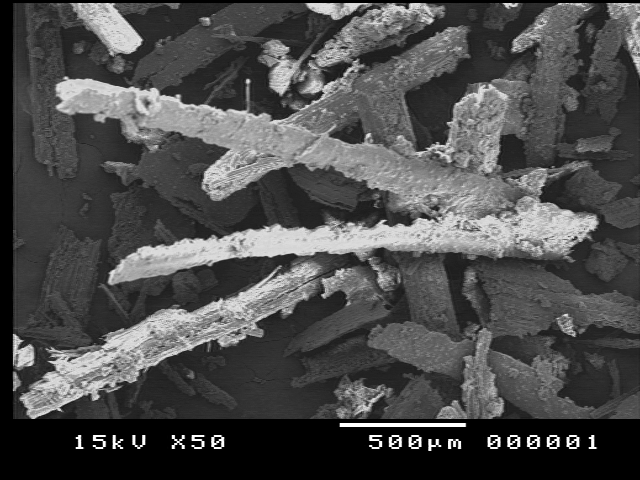

Supplement: Supplementary file 3 — Multimedia component 3 [file mmc3.zip › dib_103971_1-1_mmc3.bmp]

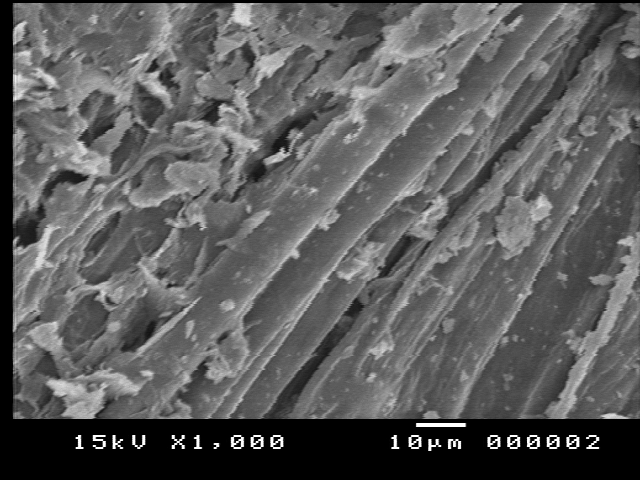

Supplement: Supplementary file 4 — Multimedia component 4 [file mmc4.zip › dib_103971_1-2_mmc4.bmp]

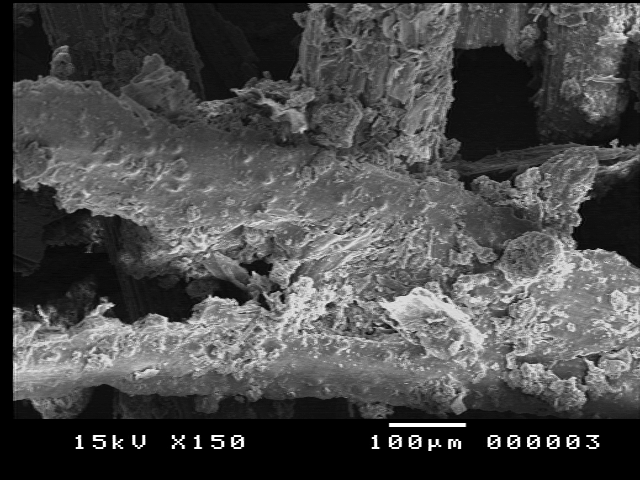

Supplement: Supplementary file 5 — Multimedia component 5 [file mmc5.zip › dib_103971_1-3_mmc5.bmp]

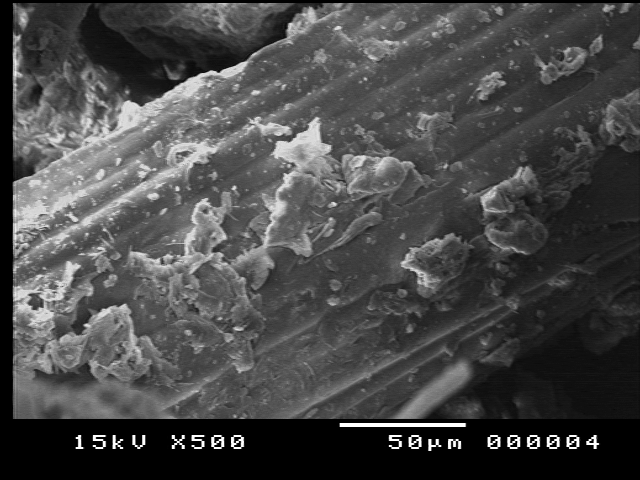

Supplement: Supplementary file 6 — Multimedia component 6 [file mmc6.zip › dib_103971_1-4_mmc6.bmp]

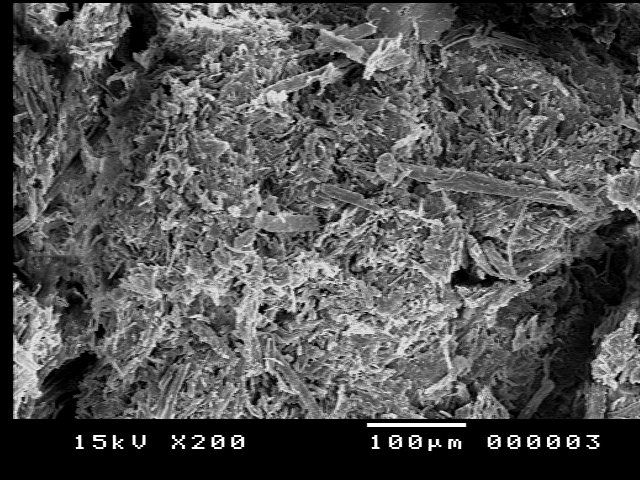

Supplement: Supplementary file 7 — Multimedia component 7 [file mmc7.zip › dib_103971_2_mmc7.bmp]

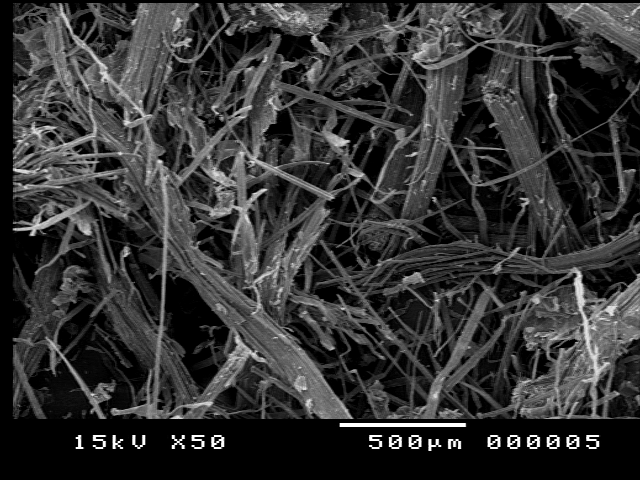

Supplement: Supplementary file 8 — Multimedia component 8 [file mmc8.zip › dib_103971_2-1_mmc8.bmp]

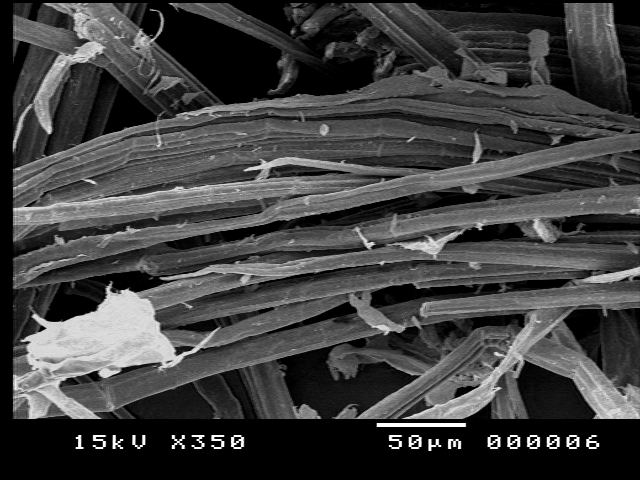

Supplement: Supplementary file 9 — Multimedia component 9 [file mmc9.zip › dib_103971_2-2_mmc9.bmp]

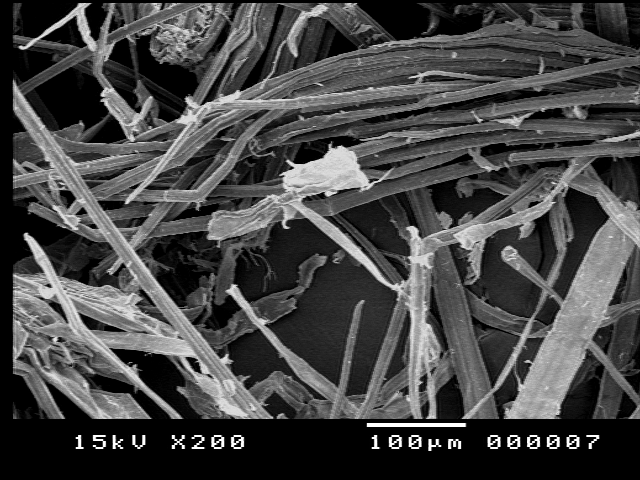

Supplement: Supplementary file 10 — Multimedia component 10 [file mmc10.zip › dib_103971_2-3_mmc10.bmp]

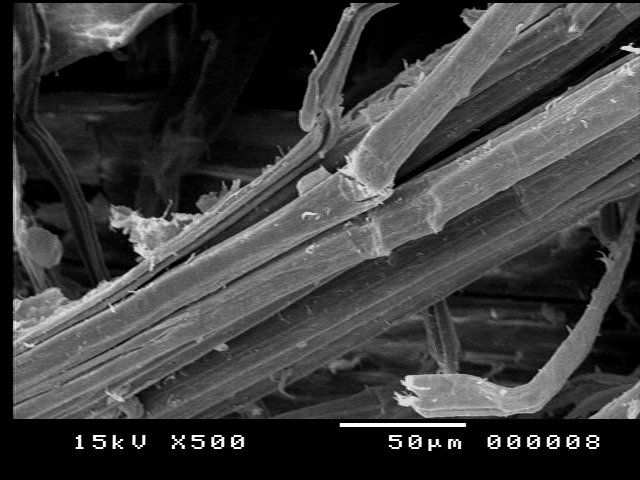

Supplement: Supplementary file 11 — Multimedia component 11 [file mmc11.zip › dib_103971_2-4_mmc11.bmp]

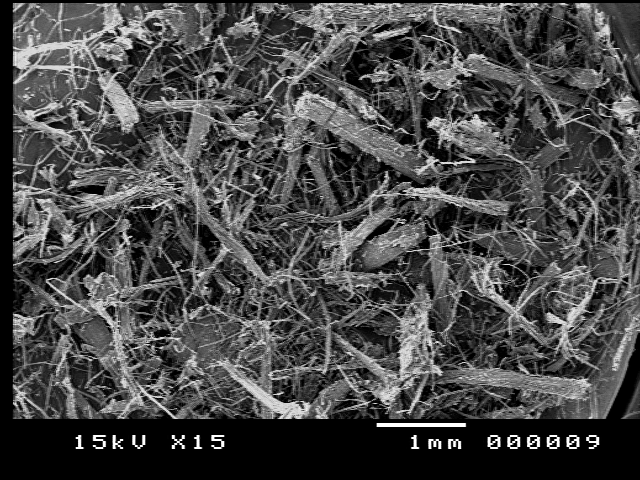

Supplement: Supplementary file 12 — Multimedia component 12 [file mmc12.zip › dib_103971_2-5_mmc12.bmp]

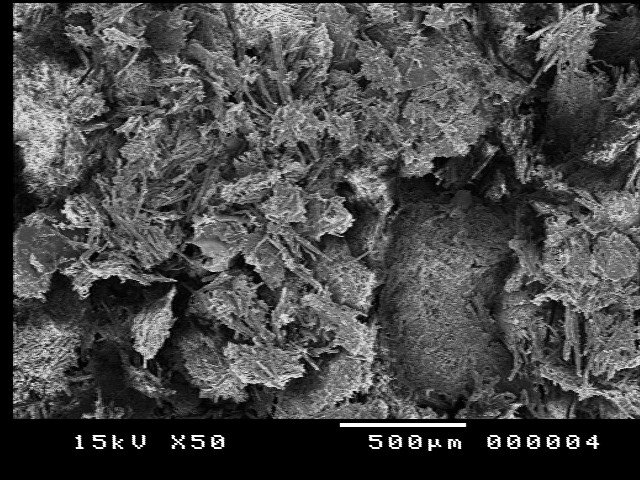

Supplement: Supplementary file 13 — Multimedia component 13 [file mmc13.zip › dib_103971_3_mmc13.bmp]
